# Supplementary material for: Platelet-Rich Plasma (PRP) and Adipose-Derived Stem Cell (ADSC) Therapy in the Treatment of Genital Lichen Sclerosus: A Comprehensive Review
Source: Int J Mol Sci. 2023 Nov 9;24(22):16107. doi: 10.3390/ijms242216107 (PMC10671587; doi:10.3390/ijms242216107)
Supplement: Supplementary file 1 [file ijms-24-16107-s001.zip › ijms-2690942-supplementary.pdf]

Supplementary Figure S1.

Supplementary Figure S1 PRISMA DIAGRAM

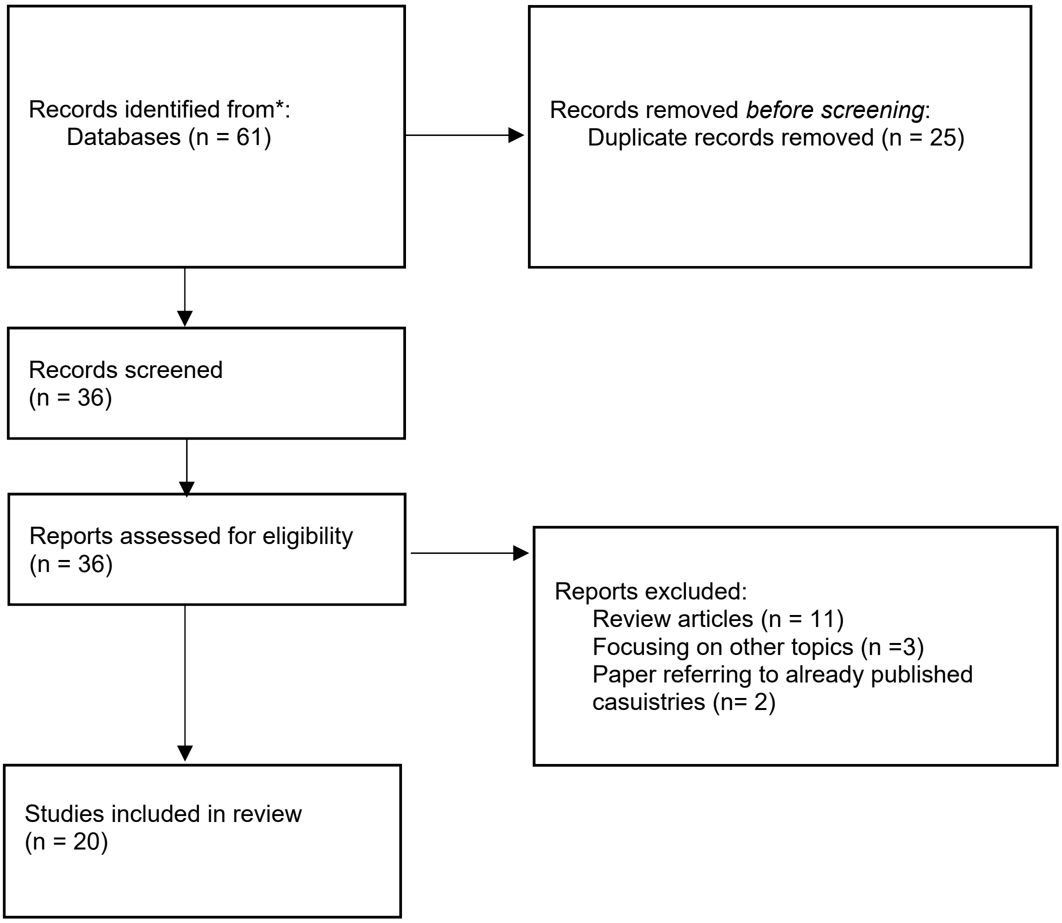

Figure S1. Flow-Chart indicating the main publications containing original data (RCT, case series or case reports) focusing on the use of ADSCs and PRP in the treatment of LS. Main Findings are summarized in Table 1 of the manuscript.

Supplementary Table S1.

Table S1. ADSC and PRP-based treatment protocols for genital LS

AUTHOR	Y	Therapy	control group	age	n° tt	interval	FUP m	OUTCOMES
Almadori	2020	ADSC			1		12.9	pain, burning, itching, soreness, HADS, RAS, WMQR, VASS, FSFI, FSDS
Behnia-Willison	2016	PRP		60 (22-88)			24	soreness, discomfort, dyspareunia, pain, itching, Australian Pelvic Floor Questionnaire
Boero	2015	ADSC						DLQI, FSFI, mucocutaneous trophism
Casabona	2010	ADSC+PRP		(27-62)	at least 1	3m		pain, burning, itching
Casabona	2017	PRP		49.96 (+-11.32)	(2-10)		17.6 (+-5.63)	phimosis, splitting, inflammation, synechiae, meatus stenosis, pain, burning, itching, DLQI, IGA, Liekert
Casabona	2023	ADSC+PRP						CSS, DLQI, Skindex, FSFI
Cohen	2019	ADSC+PRP						atrophy
Franic	2018	PRP		38	2	2m	3	ICIQ-VS, FSFI
Gutierrez-Ontalvilla	2022	ADSC+PRP	Y (topical CS)				12	erosions, fissures, stenosis, leukoderma, pain, burning, itching, dyspareunia
Goldstein	2016	PRP			2	6w	3	IGA, VAS for pruritus and burning, histological inflammation scale 0-3
Goldstein	2019	PRP	Y (placebo)	52.6	2	6w		CSS, histological inflammation scale 0-3
Kim	2017	ADSC+PRP		67				pruritus, irritation, volume and tone of the labia

Medina Garrido	2023	PRP			3		12	CSS
Monreal	2020	ADSC					24	modified vulvo-vaginal symptoms questionnaire
Navarrete	2020	PRP			6.8	8w	18	DLQI, IGA
Onesti	2016	ADSC			2	3m		trphism, pain
Tedesco	2019	PRP			3	15 d		pain, burning, itching, dyspareunia
Tedesco	2020	ADSC+PRP	Y (ADSC)	(43-78)				DLQI
Tedesco	2021	PRP			1		1	skin temperature (°C)
Tedesco	2022	PRP			3	15 d	6	pain, burning, itching, dyspareunia
